# Seroma change during magnetic resonance imaging-guided partial breast irradiation and its clinical implications

**DOI:** 10.1186/s13014-017-0843-7

**Published:** 2017-06-20

**Authors:** Seung Hyuck Jeon, Kyung Hwan Shin, So-Yeon Park, Jung-in Kim, Jong Min Park, Jin Ho Kim, Eui Kyu Chie, Hong-Gyun Wu

**Affiliations:** 1 Department of Radiation Oncology, Seoul National University College of Medicine, Seoul, Republic of Korea; 2 Department of Radiation Oncology, Seoul National University Hospital, Seoul, Republic of Korea; 3 Biomedical Research Institute, Seoul National University Hospital, Seoul, Republic of Korea; 4 Institute of Radiation Medicine, Seoul National University Medical Research Center, Seoul, Republic of Korea

**Keywords:** Breast cancer, Partial breast irradiation, Adaptive radiotherapy, Seroma

## Abstract

**Background:**

To investigate the patterns of post-lumpectomy seroma volume (SV) change and related clinical factors to determine the benefits of adaptive planning in magnetic resonance imaging (MRI)-guided partial breast irradiation (PBI).

**Methods:**

MRI data obtained from 37 women with early breast cancer acquired at simulation and at the 1st, 6th, and 10th fractions were analyzed. The planning target volume (PTV) was defined as unequal margins of 10–15 mm added according to the directional surgical margin status of each seroma. Treatment was performed using a 0.35 T MRI-guided radiotherapy system. Univariate analysis was performed to assess the correlations between SV change rate and clinical factors. Seroma and PTV for adaptive planning were based on the images obtained at the 6th fraction.

**Results:**

The average time intervals between surgery-simulation, simulation-1st, 1st-6th, and 6th-10th fractions were 23.1, 8.5, 7.2, and 5.9 days, respectively. Of the 37 patients, 33 exhibited decreased SV over the treatment period. The mean SV of these 33 patients decreased from 100% at simulation to 60, 48, and 40% at each MRI scan. In most cases (26/33), the logarithm of SV was inversely proportional to the elapsed time from surgery (*R* ^2^ > 0.90, Pearson’s correlation test). The volume of spared normal tissue from adaptive radiotherapy was proportional to the absolute change in SV (*R* ^2^ = 0.89, Pearson’s correlation test).

**Conclusion:**

Seromas exhibit exponential shrinkage over the course of PBI. In patients receiving PBI, frequent monitoring of SV could be helpful in decision-making regarding adaptive planning, especially those with a large seroma.

## Background

Radiotherapy is an effective modality for reducing the recurrence rate and breast cancer mortality after breast-conserving surgery [1, 2]. Based on the spatial pattern of recurrence, partial breast irradiation (PBI) can be introduced as an alternative to conventional whole-breast irradiation followed by boost in selected patients with early breast cancer, aiming for a short treatment time and reduced toxicity. Several randomized trials and recent meta-analyses have reported comparable oncologic outcomes with PBI [3–6]. As more evidence is accumulated, the scope of indications for PBI is expanding [7].

In PBI, defining the optimal target volume is crucial to thoroughly cover the area at risk and to simultaneously minimize toxicity. The target volume of PBI is defined as tissues surrounding the tumor bed [8]. After superficial closure of the excision cavity, the tumor bed often appears as a seroma [9, 10], which makes target definition more clear and consistent. Seroma volume (SV) is well-known to typically shrink with time [11–17]. Therefore, an ideal target volume should differ in each PBI fraction. Several studies have reported the benefits of adaptive planning of tumor bed boost concomitant with or following whole-breast irradiation [18–21], and reducing the treatment volume by replanning in PBI is also likely to improve normal tissue protection.

In light of the seroma definition and re-imaging for adaptive radiotherapy, magnetic resonance imaging (MRI) has several advantages over computed tomography (CT). MRI provides improved visibility of the seroma [22], facilitating both the delineation of the tumor bed and the monitoring of its volume, whereas breast parenchyma may prevent the clear visualization of seromas in CT. The lack of radiation exposure is another advantage of MRI. Therefore, the MRI-guided technique is believed to be superior to conventional CT-based radiotherapy in PBI. However, because MRI is not routinely exploited in breast cancer, to date no studies have focused on MRI-based SV changes during PBI. Therefore, we conducted a retrospective analysis to investigate the patterns of SV change. Our objectives were to build a simple mathematical formula to explain SV change, identify clinical factors associated with the patterns, and propose the benefits of replanning halfway through the course of treatment.

## Methods

### Patient selection

In our institution, indications of PBI are as follows: age 50 years or more, invasive ductal carcinoma, pathologic stage T1N0, luminal A subtype, adequate surgical margin, and no chemotherapy. From October 2015 to July 2016, 55 patients received PBI and 38 patients developed a well-defined post-lumpectomy seroma (seroma with cavity visualization score, which was defined by Landis et al. [23], of 4 or 5), including one patient with a seroma lying from the primary site to an axillary region, which was, in part, believed to be generated from a sentinel node biopsy and was thus excluded from the analysis. Seroma aspiration was not performed in any patient. The final analysis was performed with data derived from 37 patients. The Central Review Board of Seoul National University Hospital approved the entire course of this study.

### Treatment and MRI scanning

Radiotherapy was started no more than 6 weeks after lumpectomy. Patients underwent both CT and MRI on the same day, as part of the simulation. Patients were scanned in the supine position using a custom vacuum-lock bag for arm elevation, knee support, and a body coil on the chest for MRI acquisition. The simulation MRI was performed with ViewRay (MRIdian, Oakwood Village, OH, USA) equipped with three ^60^Co sources and a 0.35 T MRI device. Ten fractions (38.5 Gy, one fraction daily) were prescribed to all patients and were delivered by the same system. It was recommended that patients sustain shallow respiration during simulation and treatment. PBI was initiated 1 week or more following simulation. All 10 fractions were delivered following MRI acquisition and after correcting setup errors. Spatial resolution of MRI at simulation and treatment was 1.5 x 1.5 x 1.5 mm.

### Contouring and analyses

All MRI scans obtained from the ViewRay system were imported into Eclipse System (Varian Medical System, Palo Alto, CA, USA) for contouring and volume calculation. Among total of 11 MRI sets for each patient, four MRI sets obtained in approximately 1-week intervals (i.e. simulation, and 1st, 6th, and 10th fractions) were analyzed to avoid misinterpretations caused by intrapersonal variability, which could be greater than the daily SV difference.

Structure delineation was conducted by one author (Jeon SH). Contouring for one patient was performed on the same day to minimize intrapersonal variation. Changes in SV were classified into three categories: decrease, increase, and stationary. Considering the intrapersonal contouring variability, -3% and +3% of change was defined as the threshold for a decrease and increase of SV, respectively. The SV was considered stationary when the difference was between -3% and +3%; the cutoff value was based on our experience regarding intrapersonal variability.

According to our institution’s policy, the clinical target volume (CTV) was defined as unequal expansion of 10–15 mm from seroma. An expanded margin of a certain direction was determined according to the resection margin status: a 10 mm expansion to the directions with a 10 mm or larger resection margin, and a 15 mm expansion with less than a 10 mm resection margin. The CTV was limited to 3 mm below the skin surface and chest wall/pectoral muscles. CTV modification on the superficial region was allowed when the seroma was close to the skin surface. Planning target volume (PTV) was identical with CTV, because application of differential margin may obviate the need of larger margin and we performed image-guided therapy in every fraction. Initial and adapted PTVs were contoured on the simulation and 6th fraction images, respectively, and their difference (ΔPTV_sim-6th_) was considered to be the volume of spared normal tissue from adaptive planning.

### Statistical analysis

All statistical analyses were performed using SPSS (version 22.0 SPSS Inc., IBM). Quantitative data are expressed as the mean ± standard deviation. Student’s *t*-test was used to assess the mean difference. The Kolmogorov-Smirnov test was used to determine whether continuous variables follow a normal distribution and Pearson’s correlation analysis was performed to investigate the relationship between two continuous variables. All statistical analyses were considered significant when *p* <0.05.

## Results

### Patient characteristics

Clinical characteristics of the included patients are detailed on Table 1. The median age of patients was 57 years with a median body mass index of 25.5 kg/m^2^. The patient population was equally distributed between left- and right-sided tumors (19 and 18 patients, respectively). The seroma was located in the upper-outer, upper-inner, lower-outer, and lower-inner quadrant of the breast in 12, 18, 6, and 1 patients, respectively. The time interval from lumpectomy to simulation was 23.1 ± 5.4 days.

**Table 1:** Clinical characteristics of the included 37 patients

Characteristic	N (%)
Total	37
Age	
50–59 years	25 (67.6%)
≥ 60 year	12 (32.4%)
Pathologic T stage	
T1a	3 (8.1%)
T1b	16 (43.2%)
T1c	18 (48.6%)
Histologic grade	
1	15 (40.5%)
2	20 (54.1%)
3	2 (5.4%)
Resection margin	
1–9 mm	36 (97.3%)
≥ 10 mm	1 (2.7%)
Hormone Receptor	
Positive	37 (100.0%)
Negative	0 (0.0%)
HER-2	
Positive	0 (0.0%)
Negative	37 (100.0%)
Ki-67	
< 15%	37 (100.0%)
≥ 15%	0 (0.0%)

### Patterns of SV change and mathematical representation

**Fig. 1:**
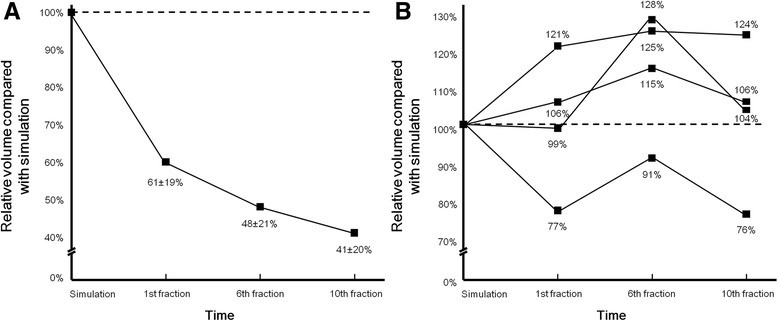
**a** Overall pattern of SV change in the 33 patients without an increase in seroma and (**b**) individual patterns of SV change in the four patients with an increase in seroma

The initial SV of 37 patients ranged from 1.26 cm^3^ to 82.33 cm^3^ (median, 14.80 cm^3^). The time intervals from simulation to the 1st fraction, from the 1st fraction to the 6th fraction, and from the 6th fraction to the 10th fraction were 8.5 ± 2.6, 7.2 ± 0.4, and 5.9 ± 0.8 days, respectively. Four patients (10.8%) had a seroma that increased during at least one interval after simulation, three of which developed a larger seroma during treatment compared to simulation. The other 33 patients exhibited a decreasing or stationary pattern of SV throughout the entire period. The overall pattern of SV changes in the 33 patients is shown in Fig. 1a. The individual patterns of four patients with an increasing seroma are detailed in Fig. 1b. Table 2 lists the number of patients with each pattern according to the time period. All analyses below were performed on the data obtained from the 33 patients.

**Table 2:** Patterns of seroma volume change according to the time interval

Seroma volume change	Number of patients (*n* = 37)		
	Simulation-1st fraction (mean, 8.5 days)	1st -6th fraction (mean, 7.2 days)	6th -10th fraction (mean, 5.9 days)
Increase	2	3	0
Stationary	1	5	5
Decrease	34	29	32

To build a mathematical model for SV change, we examined the correlation between SV change and SV itself. The SV change ratio per day (SVR) was calculated, assuming that SV decreases at the same rate. Using Pearson’s correlation test, we found no association between SV_sim_ and SVR_sim-1st_, SV_1st_ and SVR_1st-6th_, or SV_6th_ and SVR_6th-10th_. Because SV had no effect on relative SV decrease rate, the absolute decrease of SV was proportional to SV, and was therefore used to build a mathematical representation. We generated the following equation: SV_d_ = SV_0_ · exp(-*kd*), where *d* is the elapsed time (day) from the initial point of time, *k* is a constant related to shrinking speed, SV_0_ is the initial SV, and SV_d_ is the SV on the *d* ^th^ day. By changing the equation (as shown below), we were able to apply a linear correlation analysis.

ln (SV_d_/SV_0_) = -*kd*

**Fig. 2:**
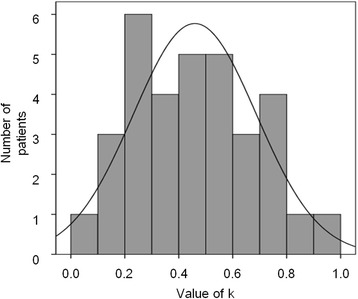
Distribution of the constant *k* in 33 patients with a seroma (without increase). The corresponding normal distribution (mean, 0.046; standard deviation, 0.023) curve is also depicted

By applying Pearson’s correlation test, we determined the value of *k* and the coefficient of correlation, or R^2^, for each patient, where higher *k* and R^2^ values indicate faster reduction of the seroma and better fitness to the equation, respectively. Figure 2 shows the distribution of *k* in the 33 patients. We confirmed that the distribution followed a normal distribution (Kolmogorov-Smirnov test), with a mean and standard deviation of 0.046 and 0.023, respectively. The proportions of patients with an R^2^ value greater than 0.8 and 0.9 were 90.9% (30/33) and 78.8% (26/33), respectively.

### Effects of clinical factors

We analyzed associations between SV change and all clinical factors, including age, body mass index, location of the seroma, and radiotherapy. The time interval from surgery to the 10th fraction was analyzed as a substitute for radiotherapy, because a longer interval represents less influence from radiotherapy. The median value of each continuous variable was set as the cutoff point, dividing the patients into two groups for comparison. Table 3 displays the *k* values and reveals no associations with clinical factors, except body mass index (*p* = 0.02) with SV reduction rate.

**Table 3:** Association between clinical factors and constant, k

Variable	n	k	^a^p
Age			
< 57 years	16	0.048 ± 0.022	0.55
≥ 57 years	17	0.044 ± 0.024	
Body mass index			
< 25 kg/m^2^	14	0.057 ± 0.023	0.02
≥ 25 kg/m^2^	19	0.038 ± 0.020	
Location (upper vs. lower)			
Upper	26	0.049 ± 0.022	0.11
Lower	7	0.034 ± 0.024	
Location (outer vs. inner)			
Outer	18	0.049 ± 0.023	0.43
Inner	15	0.042 ± 0.023	
Surgery-10th fraction interval			
< 43 days	16	0.053 ± 0.025	0.09
≥ 43 days	17	0.039 ± 0.019	

^a^Student’s *t*-test

### Normal tissue sparing and SV change

**Fig. 3:**
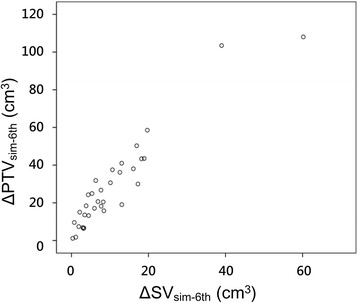
Change in PTV from adaptive planning with respect to the absolute SV difference between simulation and the 6th fraction

The volume of the initial PTV ranged from 32.3 cm^3^ to 209.5 cm^3^ (median, 69.5 cm^3^) and the adapted PTV ranged from 18.7 cm^3^ to 123.9 cm^3^ (median, 51.0 cm^3^). The volume of adapted PTV was significantly smaller than that of the initial PTV (*p* < 0.01, Student’s *t*-test). Figure 3 displays the proportional correlation between decreased SV from simulation to the 6th fraction, or ΔSV_sim-6th_, with ΔPTV_sim-6th_ (*p* < 0.01, *R* ^2^ = 0.890, Pearson’s correlation test).

## Discussion

Using data from multiple MRIs obtained before and after PBI, we found that the patterns of SV change can be described using a mathematical representation. Although numerous studies have reported that the patterns of seroma change over time, the current study utilized new methodology with a more specific group of patients. Unlike most previous studies, which elaborated on seroma changes during whole-breast irradiation, we examined patients receiving PBI. Therefore, we focused on SV change within a relatively shorter time period compared to other studies, most of which compared images with long intervals. Additionally, adjuvant chemotherapy is not allowed in PBI, making the time interval from lumpectomy to radiotherapy shorter. Kader et al. [12] concluded that seroma change stabilizes at 9–14 weeks after surgery. In patients receiving PBI, however, radiotherapy ends before 8 weeks after surgery (less than 6 weeks before initiation plus 2 weeks of treatment). Hence, all patients receiving PBI should have a seroma with dynamic changes; thus, we investigated this short-term and dynamic period. Additionally, MRI is believed to provide more clear visualization of seroma compared to CT- or fiducial-based methods. Yue et al. [17] reported SV changes during PBI using gold fiducial markers and daily kV images. Despite the lack of reports comparing MRI and radio-opaque markers directly, MRI is believed to be more accurate in displaying post-lumpectomy seromas, because surgical clips define only several points on the tumor bed. To our knowledge, this is the first study to examine SV change using MRI.

Consistent with previous analyses, we demonstrated that the seroma reduction rate is inversely correlated with the time elapsed from surgery [15]. Seroma fluid absorption is believed to contribute to its reduction, although some researchers argue that the generation of granulation tissues is an underlying mechanism [24]. The increment of SV, however, may involve completely different dynamics (e.g., fluid leakage or inflammation). Therefore, we excluded patients with an increasing seroma from subsequent analyses. The correlation between SV and its reduction has been demonstrated in previous studies [14, 16]. Our data revealed this correlation as well. Based on this observation, we devised a formula to describe SV changes over time. An exponential shrinkage pattern describes the proportional relationship between SV and SVR at any time. With the exception of four patients with an increase in seroma, the pattern of SV change fitted well (*R* ^2^ > 0.90) into the formula in 78.8% (26/33) of patients.

Yue et al. reported an average half-life for seroma shrinkage of 15 days in patients treated with PBI [17]. With the mean *k* = 0.046, the average half-life calculated using our formula is 15.0 days, which is the same as that obtained by Yue et al. In the clinic, however, seroma is still observed in some patients after several months following surgery. Our equation may not explain SV change long after surgery. Additionally, the immediate postoperative status of seroma change may differ in its mechanism and pattern, because it can be influenced by early elements such as acute inflammation. Although we cannot assure the suitability of our mathematical model throughout the entire lifespan of seroma, it is evident from the data that our model explains the SV change during the period of interest in the majority of cases. We believe that the significance of our formula is its ability to predict the upcoming SV change pattern using data from the early course, thereby aiding in the early selection of candidates for adaptive radiotherapy.

Several factors (e.g., biologic microenvironment, postoperative complication, and external forces) are assumed to be associated with seroma formation and change [24]. In our analysis, a low body mass index was a statistically meaningful factor associated with fast seroma shrinkage. This correlation may have resulted from an external force on the seroma that may be affected by the amount of surrounding breast tissue. Our study did not find a significant association between radiotherapy and SV change. Yang et al. [13] suggested that radiotherapy hinders seroma reduction in patients receiving whole-breast irradiation; in their study, the time intervals between surgery and the start of radiotherapy (mean, 34 days) and the two CTs (mean, 23 days) were much longer than those used in our study. It is likely that the time period of PBI is not sufficient to reveal the effects of radiotherapy to SV change. We conclude that the clinical factors examined herein are not sufficient for the estimation of seroma shrinkage rate. Therefore, we believe that every patient with a seroma should be monitored for successful adaptive planning.

We examined the potential benefits of adaptive planning applied from the 6th fraction of PBI. The decision whether or not to replan should be made on or before the day of the 6th fraction. Assurance that the seroma will not increase thereafter is a prerequisite for safe adaptive radiotherapy. As shown in Table 1, all patients did not exhibit an increase in the last period (from the 6th to the 10th fraction), suggesting that adaptive planning on the day of the 6th fraction does not threaten target coverage by an increase in seroma. Conversely, replanning is imperative to prevent a geographic miss whenever the seroma at treatment outgrows the initial seroma. To screen for an increase in SV, frequent monitoring of the seroma, especially during the early phase of treatment, may be helpful for safe PBI.

In PBI, patients with a decrease in seroma are expected to exhibit an improvement in dosimetric parameters after replanning. Nonetheless, we believe that adaptive radiotherapy should be applied in selected patients with significant dosimetric benefits. The criteria of replanning are within the discretion of clinicians and may differ according to treatment techniques, as several modalities are practicable in PBI [25, 26]. Chen et al. [20] reported significant breast tissue sparing when the SV decrease is larger than 35%, and Mohiuddin et al. [21] recommended replanning when the patient experienced ≥5 cm^3^ and ≥25% decreases in SV. However, these studies investigated adaptive planning in tumor bed boost irradiation following whole-breast irradiation. Because SV and PTV demonstrated a close relationship in the present study (see Fig. 3), we agree that absolute SV change can play a role as an indicator for adaptive planning. Hence, frequent monitoring of seroma may be more crucial for patients with a large seroma.

The limitations of the current analysis include small number of patients, limited role of seroma on target volume delineation, and absence of dosimetric data. After closed cavity surgery, seroma is not visible on postoperative imaging. In our institution, 17 (31%) out of 55 patients showed no visible seroma on postoperative MRI and our results cannot be applied to these patients. In addition, we did not demonstrate dosimetric advantages of adaptive planning, which may suggest practical guideline. Therefore, further studies regarding the dosimetric improvement in adaptive planning in PBI are needed to establish the selection criteria for replanning.

In summary, we described the patterns of SV change using MRI and proposed a mathematical representation for SV prediction. Because the current study elaborates on SV changes taking place before approximately 2 months after lumpectomy, the results should be applied with caution to patients receiving conventional whole-breast irradiation, especially after chemotherapy.

## Conclusions

After lumpectomy, seromas exhibit exponential shrinkage over time. In patients receiving PBI, frequent monitoring of SV could be helpful in decision-making regarding adaptive planning, especially those with a large seroma.
